# Does the Mother or Father Determine the Offspring Sex Ratio? Investigating the Relationship between Maternal Digit Ratio and Offspring Sex Ratio

**DOI:** 10.1371/journal.pone.0143054

**Published:** 2015-11-17

**Authors:** Tae Beom Kim, Jin Kyu Oh, Kwang Taek Kim, Sang Jin Yoon, Soo Woong Kim

**Affiliations:** 1 Department of Urology, Gachon University Gil Hospital, Incheon, Republic of Korea; 2 Department of Urology, Seoul National University Hospital, Seoul, Republic of Korea; University of Vienna, AUSTRIA

## Abstract

**Objective:**

In mammals, high parental testosterone levels present around the time of conception are thought to skew offspring sex ratio toward sons. The second to fourth digit ratio (digit ratio) is now widely accepted as a negative correlate of prenatal testosterone. Thus, we investigated the association between digit ratio and offspring sex ratio.

**Methods:**

A total of 508 Korean patients (257 males and 251 females) less than 60 years old who had one or more offspring were prospectively enrolled. The lengths of the 2nd and 4th digits of the right hand were measured by a single investigator using a digital vernier calliper. Next, the patients’ lifetime offspring birth sex ratios were investigated.

**Results:**

Maternal (rather than paternal) digit ratio was significantly associated with the number of sons (r = -0.153, p = 0.015), number of daughters (r = 0.130, p = 0.039), and offspring sex ratio (r = -0.171, p = 0.007). And, the maternal digit ratio was a significant factor for predicting offspring sex ratio (B = -1.620, p = 0.008) on multiple linear regression analysis. The female patients with a lower digit ratio (< 0.95) were found to have a higher offspring sex ratio (0.609 versus 0.521, p = 0.046) compared to those with a higher digit ratio (≥ 0.95). Furthermore, females in the low digit ratio group have a probability 1.138 greater of having sons than females in the high digit ratio group.

**Conclusions:**

Maternal digit ratio was negatively associated with offspring sex ratio. Females with a lower digit ratio were more likely to have more male offspring compared to those with a higher digit ratio. Thus, our results suggest that the sex of offspring might be more influenced by maternal rather than paternal factors.

## Introduction

The second to fourth digit ratio (digit ratio) is known to be sexually dimorphic; females have larger digit ratios than males [1–4]. This ratio is primarily determined during fetal development [5–8] and changes little after sexual maturation [8]. This gender-associated difference may be influenced by changes in prenatal steroid concentrations [1,5]. Prenatal testosterone concentrations have been demonstrated to cause sex differences in digit ratio [9,10]. The direction of change of the digit ratio depends on the gestational timing and duration of fetal testosterone exposure [9,10]. Thus, digit ratio is now widely accepted as a negative correlate of prenatal testosterone around about the end of the first trimester [1,5,11,12].

However, there is no simple understanding of fetal testosterone exposure from determining adult digit ratio. Hollier et al. [13] reported that adult digit ratio is not related to umbilical cord androgens or estrogens concentrations at late gestation. Furthermore, there is probably not a strong correlation between digit ratio and adult testosterone levels. However, the waist-to-hip ratio (WHR) in women is positively correlated with serum levels of testosterone and negatively with women's digit ratio. Furthermore, the WHR of mothers has been reported to correlate negatively with the digit ratio of their children [14]. This suggests that women with genes for high testosterone have children with low digit ratio.

In mammals, high parental testosterone levels present during conception have been proposed to skew the offspring sex ratio toward sons [15,16]. In addition to circulating testosterone concentration, high maternal estrogen levels have been reported to correlate with female-biased offspring sex ratios among gray mouse lemurs [17]. To date, there are three papers that have examined links between digit ratio and offspring sex ratio. Manning et al. reported that both men and women with lower digit ratios had significantly higher offspring sex ratios (i.e., more male offspring) in a large (n = 456) cross-cultural study (Spanish, English, and Jamaican) [18]. This paper was followed by that of Helle and Lilley who reported that in a sample of Finnish women, maternal digit ratio could not predict lifetime offspring sex ratios [19]. However, Ventura et al. reported digit ratio data from 106 mothers and their children and found mothers with low digit ratio tended to have sons [20].

Up to this point, data indicating the relationship between digit ratio and offspring sex ratio are inconsistent and controversial. Therefore, the purpose of the present study was to investigate the association between digit ratio and offspring sex ratio.

## Materials and Methods

### Study participant selection

Of the patients that were hospitalized for urological surgery at a single tertiary academic center, a total of 508 (257 males and 251 females) Korean patients less than 60 years old who had one or more offspring were prospectively enrolled in the study. Ethical approval (approval number: GBIRB2013-88) was obtained from the Institutional Review Board (IRB) of Gachon University Gil Hospital (Incheon, Republic of Korea). All participants signed an informed consent. Patients with a history of induced abortion that artificially affected the offspring sex ratio were excluded. In addition, patients with a history of arthritis (e.g., rheumatoid arthritis), as well as those who had fingers amputated, were excluded.

### Digit measurement

Before assessing the patients’ offspring, the 2nd and 4th digits of the right hand were directly measured by a single investigator using a digital vernier calliper accurate to 0.01 mm. Digit length of right hand was measured on the ventral surface of the hand from the basal crease of the digit to the fingertip [1]. To standardize keeping the fingers similarly straight during measurements, fingers and hands were placed on a flat hard surface of desk. In order to reduce errors in measurement, the mean of two digit ratios calculated based on duplicate measurements were used for the analysis. For assessing repeatability of two measurements, we used the method proposed by Bland and Altman [21]. The average of differences between two digit ratios calculated based on duplicate measurements using a digital vernier calliper by a single investigator is -0.001. The standard deviation of differences between the 508 pairs of repeated measurements is 0.013. The coefficient of repeatability is twice this, or 0.026 for digital vernier caliper (Mean - 2SD = -0.027; Mean + 2SD = 0.025). This suggests that a direct measurement of digit ratio using a digital vernier calliper has acceptable repeatability [21].

### Investigation of the offspring sex ratio

Information about the patients’ lifetime offspring birth sex ratio was collected. For the present study, we defined offspring sex ratio as the proportion of sons to total offspring (offspring sex ratio = number of sons / number of total offspring).

### Statistical analysis

Relationships between study variables were analyzed using Pearson’s linear correlation. To identify significant predictive factors that influenced the offspring sex ratio, multiple linear regression analyses were performed. Student’s *t*-test and relative risk (RR) analysis were used to compare variables for the two study groups arranged according to digit ratio. To compare the offspring sex ratios between three groups arranged according to the number of total offspring, one-way analysis of variance (ANOVA) with post hoc Bonferroni test was used. Analyses were performed using SPSS 12.0 (SPSS, Chicago, IL, USA) and p-values less than 0.05 were considered statistically significant.

## Results

### Participant characteristics

The mean and median age of all subjects were 50.1 and 51.0 (range: 30.0–59.0), respectively. The mean and median number of total offspring of all 508 patients were 1.94 and 2.00 (range: 1–6), respectively. The median digit ratios of males, females, and all subjects were comparable 0.947 (range: 0.831–1.061), 0.952 (range: 0.791–1.123), and 0.947 (range: 0.791–1.123), respectively. The characteristics of all 508 patients are summarized in Table 1.

**Table 1:** Characteristics of the study population.

	Total	Males	Females	p-value*
N	508	257	251	
Age (yrs)	50.1 ± 6.7	50.1 ± 7.2	50.0 ± 6.2	0.928
Height (cm)	164.2 ± 8.5	170.6 ± 5.9	157.7 ± 5.3	<0.001
Weight (kg)	65.1 ± 11.0	70.7 ± 10.4	59.3 ± 8.4	<0.001
BMI (kg/m^2^)	24.1 ± 3.2	24.2 ± 3.1	23.9 ± 3.2	0.163
Second digit length (cm)	6.821 ± 0.464	7.039 ± 0.399	6.597 ± 0.418	<0.001
Fourth digit length (cm)	7.189 ± 0.498	7.436 ± 0.423	6.936 ± 0.439	<0.001
Digit ratio	0.949 ± 0.033	0.947 ± 0.030	0.952 ± 0.037	0.112
Number of total offspring	1.94 ± 0.69	1.89 ± 0.67	2.00 ± 0.72	0.077
Number of sons	1.04 ± 0.71	1.00 ± 0.71	1.08 ± 0.70	0.182
Number of daughters	0.90 ± 0.79	0.89 ± 0.77	0.92 ± 0.80	0.717
Offspring sex ratio	0.556 ± 0.364	0.549 ± 0.379	0.564 ± 0.349	0.656
Proportion of patients with only sons without any daughters (%)	32.9 (167/508)	34.2 (88/257)	31.5 (79/251)	0.508
Proportion of patients with one or more sons (%)	79.9 (406/508)	77.0 (198/257)	82.9 (208/251)	0.101

BMI, body mass index; Digit ratio, 2nd digit length / 4th digit length.

*p-value between males vs females.

### Correlation study

**Fig 1:**
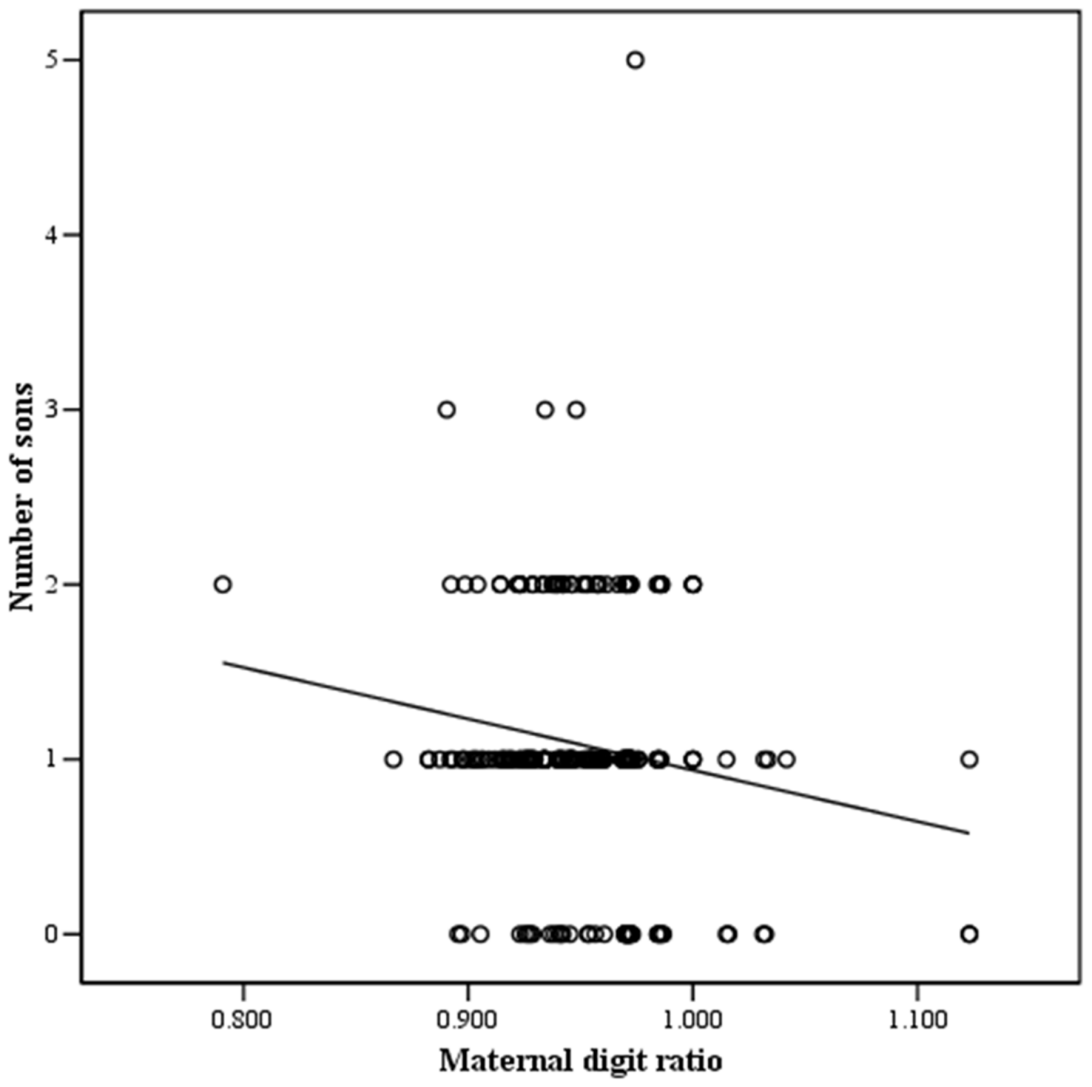
The relationship between maternal digit ratio and number of sons. Number of sons was significantly and negatively associated with maternal digit ratio. y = -2.935x + 3.873, r = -0.153, p = 0.015. y: number of sons, x: maternal digit ratio.

**Fig 2:**
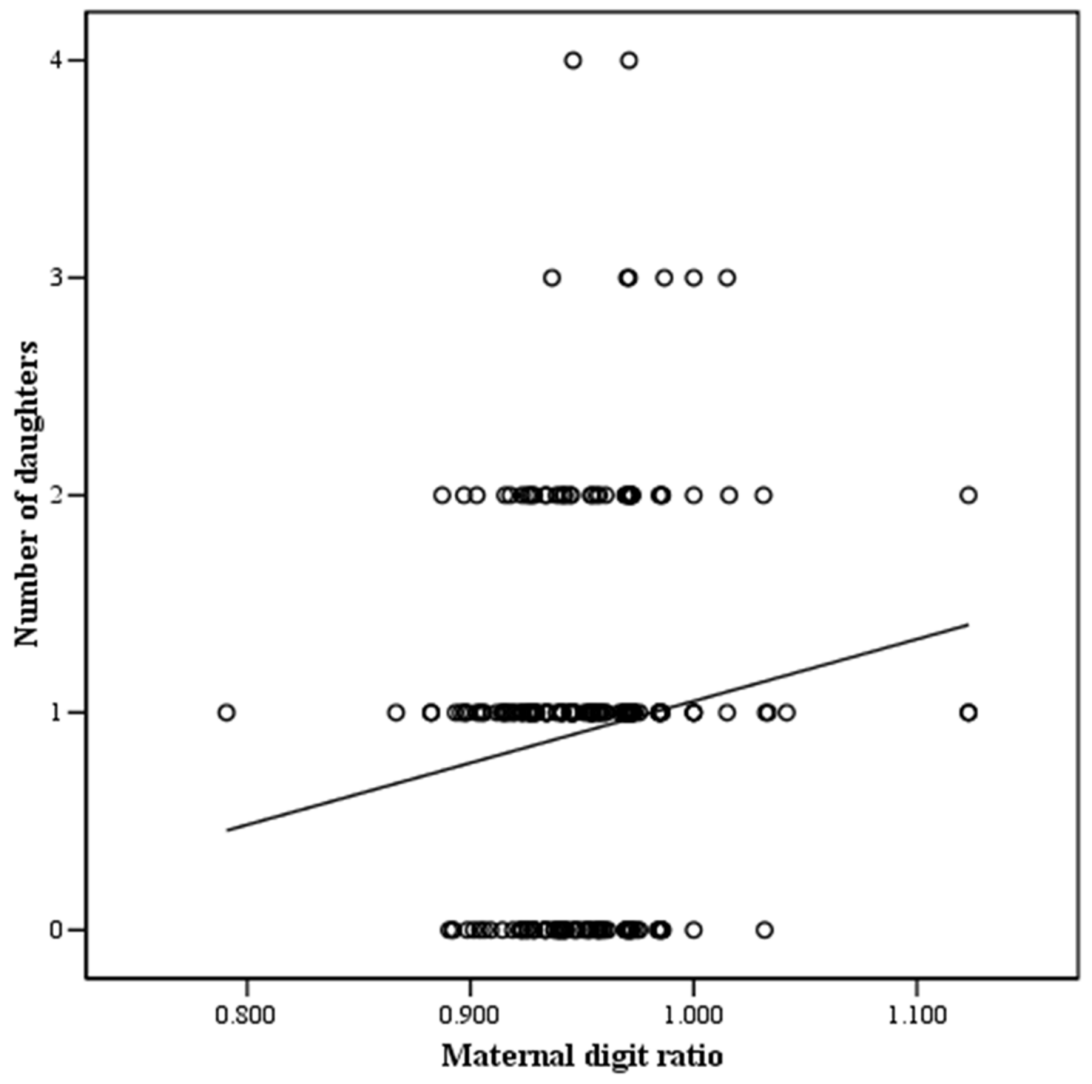
The relationship between maternal digit ratio and number of daughters. Number of daughters was significantly and positively associated with maternal digit ratio. y = 2.846x–1.793, r = 0.130, p = 0.039. y: number of daughters, x: maternal digit ratio.

**Fig 3:**
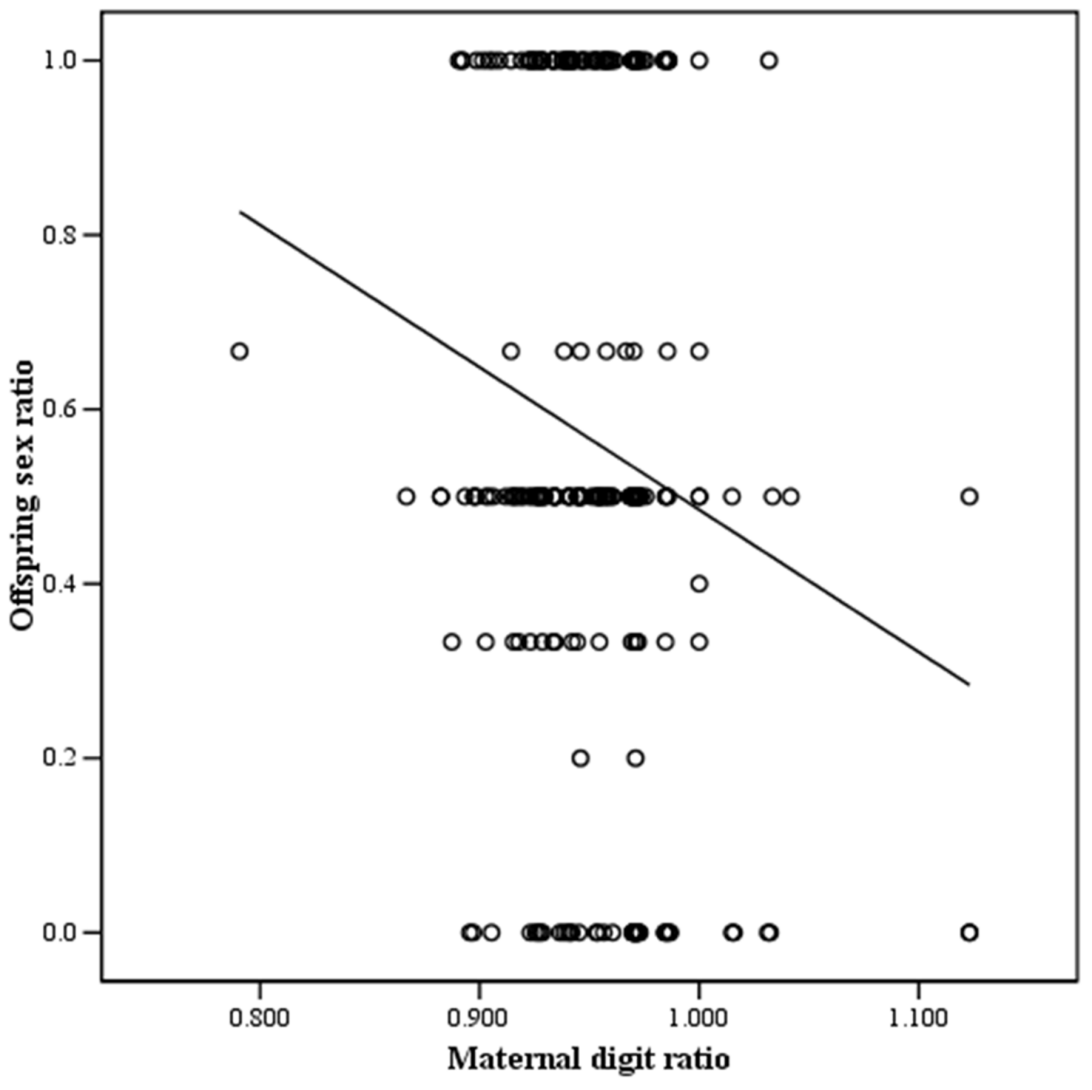
The relationship between maternal digit ratio and offspring sex ratio. Offspring sex ratio was significantly and negatively associated with maternal digit ratio. y = -1.632x + 2.117, r = -0.171, p = 0.007. y: offspring sex ratio, x: maternal digit ratio.

Among the different variables evaluated, a correlation analysis (Table 2) showed that the maternal digit ratio was significantly associated with the number of sons (r = -0.153, p = 0.015), number of daughters (r = 0.130, p = 0.039), and offspring sex ratio (r = -0.171, p = 0.007) (Figs 1–3). In contrast, male digit ratio was not correlated with the number of sons, number of daughters, or offspring sex ratio (Table 2). Results of multiple linear regression analysis (Table 3) demonstrated that the maternal digit ratio was a significant factor for predicting offspring sex ratio (B = -1.620, p = 0.008).

**Table 2:** Relationships between the study variables according to Pearson’s linear correlation analysis.

			Age	Ht	Wt	BMI	DR	T	S	D
Males	Ht	r	-0.244							
		p	<0.001							
	Wt	r	-0.223	0.485						
		p	<0.001	<0.001						
	BMI	r	-0.125	0.017	0.880					
		p	0.046	0.787	<0.001					
	DR	r	-0.040	-0.032	0.050	0.077				
		p	0.518	0.604	0.423	0.221				
	T	r	0.221	-0.095	0.040	0.097	0.027			
		p	<0.001	0.128	0.527	0.120	0.672			
	S	r	0.140	-0.102	-0.051	0.002	0.111	0.371		
		p	0.025	0.102	0.414	0.971	0.076	<0.001		
	D	r	0.062	0.012	0.081	0.082	-0.079	0.522	-0.599	
		p	0.325	0.852	0.194	0.192	0.208	<0.001	<0.001	
	SR	r	0.046	-0.054	-0.103	-0.084	0.096	-0.160	0.803	-0.875
		p	0.465	0.389	0.099	0.182	0.124	0.010	<0.001	<0.001
Females	Ht	r	-0.355							
		p	<0.001							
	Wt	r	-0.115	0.304						
		p	0.069	<0.001						
	BMI	r	0.054	-0.177	0.882					
		p	0.392	0.005	<0.001					
	DR	r	-0.033	0.055	-0.056	-0.087				
		p	0.605	0.384	0.377	0.171				
	T	r	0.233	-0.047	-0.038	-0.016	-0.005			
		p	<0.001	0.455	0.544	0.804	0.943			
	S	r	0.053	0.008	0.023	0.015	-0.153	0.367		
		p	0.401	0.902	0.719	0.813	0.015	<0.001		
	D	r	0.163	-0.049	-0.055	-0.027	0.130	0.579	-0.547	
		p	0.009	0.435	0.389	0.666	0.039	<0.001	<0.001	
	SR	r	-0.013	0.002	0.040	0.036	-0.171	-0.182	0.773	-0.841
		p	0.840	0.976	0.533	0.568	0.007	0.004	<0.001	<0.001

Ht, height; Wt, weight; BMI, body mass index; DR, digit ratio (2nd digit length / 4th digit length); T, number of total offspring; S, number of sons; D, number of daughters; SR, offspring sex ratio.

**Table 3:** Relationship between offspring sex ratio and study variables according to multiple linear regression analysis.

		B	p-value
Males	Age	0.001	0.663
	Height	0.000	0.917
	Weight	-0.004	0.143
	Digit ratio	1.302	0.101
Females	Age	-0.001	0.809
	Height	0.000	0.962
	Weight	0.001	0.659
	Digit ratio	-1.620	0.008

Multiple linear regression analyses were performed. Digit ratio, 2nd digit length / 4th digit length.

### Comparison study

The mean and median values of digit ratios of males, females, and all the patients were very close to 0.95 (Table 1). Therefore, we divided all the patients into two groups according to digit ratio cutoff of 0.95 (digit ratio < 0.95 versus digit ratio ≥ 0.95). The offspring sex ratio and proportion of patients with one or more sons (number of sons ≥ 1) among male participants were not different between the two study groups (Table 4). However, female patients in the lower digit ratio group were found to have a higher offspring sex ratio (0.609 versus 0.521, p = 0.046) and a greater proportion of individuals with one or more sons (number of sons ≥ 1) (88.4% versus 77.7%, p = 0.023) compared to those in the higher digit ratio group (Table 4).

**Table 4:** Comparisons of variables between the two study groups arranged according to digit ratio.

		DR < 0.95	DR ≥ 0.95	p-value
Males	N	141	116	
	Age (yrs)	49.8 ± 7.7	50.4 ± 6.7	0.483
	Height (cm)	170.8 ± 5.8	170.3 ± 6.0	0.487
	Weight (kg)	69.9 ± 10.0	71.6 ± 10.7	0.199
	BMI (kg/m^2^)	23.9 ± 3.0	24.6 ± 3.2	0.069
	Second digit length (cm)	6.976 ± 0.335	7.116 ± 0.455	0.006
	Fourth digit length (cm)	7.531 ± 0.359	7.321 ± 0.466	<0.001
	Digit ratio	0.926 ± 0.021	0.972 ± 0.018	<0.001
	Number of total offspring	1.89 ± 0.65	1.88 ± 0.69	0.864
	Number of sons	0.95 ± 0.71	1.05 ± 0.71	0.255
	Number of daughters	0.94 ± 0.76	0.83 ± 0.78	0.233
	Offspring sex ratio	0.525 ± 0.378	0.579 ± 0.380	0.256
	Proportion of patients with only sons without any daughters (%)	31.2 (44/141)	37.9 (44/116)	0.262
	Proportion of patients with one or more sons (%)	75.2 (106/141)	79.3 (92/116)	0.435
Females	N	121	130	
	Age (yrs)	50.8 ± 5.8	49.3 ± 6.5	0.064
	Height (cm)	157.2 ± 4.8	158.2 ± 5.7	0.161
	Weight (kg)	59.7 ± 8.6	58.9 ± 8.2	0.453
	BMI (kg/m^2^)	24.2 ± 3.2	23.6 ± 3.2	0.161
	Second digit length (cm)	6.512 ± 0.394	6.676 ± 0.426	0.002
	Fourth digit length (cm)	7.044 ± 0.425	6.836 ± 0.429	<0.001
	Digit ratio	0.925 ± 0.021	0.977 ± 0.029	<0.001
	Number of total offspring	1.98 ± 0.65	2.01 ± 0.78	0.790
	Number of sons	1.15 ± 0.64	1.02 ± 0.75	0.132
	Number of daughters	0.83 ± 0.77	0.99 ± 0.82	0.118
	Offspring sex ratio	0.609 ± 0.333	0.521 ± 0.359	0.046
	Proportion of patients with only sons without any daughters (%)	35.5 (43/121)	27.7 (36/130)	0.184
	Proportion of patients with one or more sons (%)	88.4 (107/121)	77.7 (101/130)	0.023

Student’s t-test was used to compare variables between the two study groups arranged according to digit ratio. BMI, body mass index; DR, digit ratio (2nd digit length / 4th digit length).

### Relative risk analysis

In females, the relative risk (RR) of having one or more sons in the group with digit ratio < 0.95, as compared to the ≥ 0.95 group, is 1.138, and this RR is statistically significant (95% CI: 1.017–1.274). In other words, females in the low digit ratio group have a probability 1.138 greater of having sons than females in the high digit ratio group. In males, the RR of having sons in the low digit ratio group, as compared to the high digit ratio group, is 0.948, and this RR is not statistically significant (95% CI: 0.830–1.082) (Table 5).

**Table 5:** Comparison of the probability of having one or more sons between the two study groups arranged according to digit ratio.

		No. of sons ≥ 1	No. of sons = 0	p-value	RR	95% CI
Males	DR < 0.95	106	35	0.433	0.948	0.830–1.082
	DR ≥ 0.95	92	24			
Females	DR < 0.95	107	14	0.024	1.138	1.017–1.274
	DR ≥ 0.95	101	29			

Relative risk (RR) analysis was used to compare variables of the two study groups arranged according to digit ratio. DR, digit ratio (2nd digit length / 4th digit length).

### Sub-group analysis of 394 patients with two or more children

Of 508 patients, 77.6% (394/508) have two or more children. Table 6 shows that offspring sex ratio decreases as the number of total offspring increases (Table 6). The results on Pearson’s linear correlation analysis and multiple linear regression analysis of 394 patients with two or more children are similar to those of total 508 patients (Tables 7 and 8).

**Table 6:** Comparison of offspring sex ratio according to the number of total offspring.

		Number of total offspring		
		1	2	≥ 3
Total	N	114	326	68
	Offspring sex ratio	0.667 ± 0.473* †	0.541 ± 0.327* †	0.443 ± 0.269†
Males	N	63	166	28
	Offspring sex ratio	0.667 ± 0.475* †	0.521 ± 0.345*	0.452 ± 0.261†
Females	N	51	160	40
	Offspring sex ratio	0.667 ± 0.476†	0.563 ± 0.306	0.437 ± 0.277†

p-value < 0.05 on one-way analysis of variance (ANOVA) with post hoc Bonferroni test.

*: between the patients with number of total offspring = 1 and the patients with number of total offspring = 2.

†: between the patients with number of total offspring = 1 and the patients with number of total offspring ≥ 3.

**Table 7:** Pearson’s linear correlation analysis of the patients with two or more children.

		Offspring sex ratio	
		r	p-value
Males (N = 194)	Age	0.058	0.418
	Height	-0.100	0.167
	Weight	-0.067	0.350
	Digit ratio	0.131	0.069
Females (N = 200)	Age	-0.131	0.065
	Height	0.039	0.583
	Weight	0.008	0.916
	Digit ratio	-0.149	0.035

Digit ratio, 2nd digit length / 4th digit length.

**Table 8:** Multiple linear regression analysis of the patients with two or more children.

		Offspring sex ratio	
		B	p-value
Males (N = 194)	Age	0.002	0.530
	Height	-0.004	0.425
	Weight	-0.001	0.750
	Digit ratio	1.400	0.068
Females (N = 200)	Age	-0.007	0.073
	Height	0.001	0.896
	Weight	0.000	0.905
	Digit ratio	-1.310	0.029

Digit ratio, 2nd digit length / 4th digit length.

## Discussion

Mammals usually produce approximately equal numbers of sons and daughters. For years, the belief has been that mammalian sex is randomly determined according to whether an X- or Y-chromosome-bearing spermatozoon fertilizes the oocyte. However, this belief is now being challenged by increasing evidence from field (evolutionary biologic investigations), laboratory, and clinical studies suggesting that mammalian mothers may have a decisive influence on their offspring’s sex. Additionally, researchers have suggested that ‘maternal good condition’ [22,23] and ‘maternal dominance’ [24] are related to the conception of male offspring.

### Good condition and diet

Since Trivers and Willard [22] proposed the good condition hypothesis in 1973, evolutionary biologists have found that a better maternal physical condition was significantly related to higher offspring sex ratios (i.e., more male offspring) [23]. Those choosing the good condition hypothesis looked for links in the studies of diet [25–28], and, more clearly, unsaturated fats [29], polyunsaturated fatty acids [30,31], and glucose [32].

More recently, experimental studies have demonstrated the crucial influence of pre-conceptual maternal diet on the offspring sex ratio [26,29–31,33]. A diet very high in saturated fat causes female mice to have significantly more sons than either control or insufficient fat diet females [29]. However, male mice fed similar diets (i.e., a diet very high in saturated fat) had neither more Y-chromosome-bearing sperm nor sired more male offspring than female offspring [29]. These findings suggested that the effects of diet were ‘apparent exclusively in the female’ [29]. Furthermore, a diet enriched with polyunsaturated fatty acids caused ewes to bear more male offspring [31], and a large study showed that pre-conception diet had a similar effect in humans [28].

High maternal glucose levels induced via diet before conception (pre-conceptual diets) differentially promote the development of male embryos post-conception [32,34]. Additionally, increased glucose levels in the post-conceptual uterine environment are related to the preferential development of male embryos [27,32]. These findings coincide with those from an earlier study by reproductive biologists who demonstrated that male embryos grow more rapidly than female embryos [35–39].

In humans, positive correlations also exist between maternal nutritional state and offspring sex ratio [40–44]. Recently, it has been suggested that a change in condition, rather than the maternal body condition *per se*, may be a better predictor of offspring’s sex. In other words, mothers getting an improvement in condition at the time of conception were more likely to give birth to males [15,22,45–48].

### Maternal dominance and testosterone

Most studies about maternal dominance demonstrated that more dominant females than other females in the same group are more likely to bear male offspring [23,40,49–51]. Recognizing that a tendency to dominant behavior is underpinned by testosterone [52–54], researchers who preferred the maternal dominance hypothesis sought a connection between pre-conceptual maternal testosterone concentration and offspring sex ratio [55–59]. Independently, they found that higher pre-conceptual maternal testosterone levels measured in feces [55], serum [56], and follicular fluid [57–59] were significantly associated with higher offspring sex ratios (i.e., more males than females). However, Helle et al. [56] found that paternal testosterone concentration was not associated with offspring sex ratios.

Grant et al. [58] and Garcia-Herreros et al. [59] demonstrated that the sex of bovine embryos produced by *in vitro* fertilization (IVF) may be related to the maternal preovulatory follicular testosterone levels, indicating that oocytes developing in high testosterone concentrations are more likely to be fertilized by Y-chromosome-bearing spermatozoa [33]. These findings [57,58] suggest that there may be a crucial time during the development of the zona pellucida, and that the molecular composition of the zona pellucida may be delicately influenced by high levels of follicular testosterone which make the oocyte more inclined to be fertilized by a Y-chromosome-bearing spermatozoon [33].

### Digit ratio and offspring sex ratio

One of the novel findings of the present study was that maternal (but not paternal) digit ratio was negatively associated with offspring sex ratio. And, females with a lower digit ratio were found to have a higher offspring sex ratio compared to those with a higher digit ratio. This means that the sex of offspring might be more influenced by maternal rather than paternal factors. In other words, the association of offspring sex ratio to maternal digit ratio may indicate linkage with unknown genetic traits, gestational environment or other factors related more to the mother rather than the father.

Actually, in the present study, the Pearson correlation coefficient between maternal digit ratio and offspring sex ratio was -0.171 (r = -0.171, p = 0.007), which means weak negative correlation. However, although the determination coefficient (R^2^) is very low (R^2^ = 0.030), digit ratio still remained significant after multiple linear regression analysis in females, but not in males. In other words, the significant relationship between digit ratio and offspring sex ratio appears in females but not in males. We think that this is more important in the present study.

Recently, Ventura et al. [20] measured the digit ratio of 106 newborn infants and evaluated its relationship with the maternal digit ratio and maternal testosterone levels in plasma and amniotic fluid. Maternal plasma testosterone concentrations had a negative weak correlation with the digit ratio of both male and female newborns [20]. Mothers who gave birth to sons had lower digit ratios than those who had daughters [20]. These findings indicate that maternal hormone levels play important roles in determining the offspring's sex [20]. This means that the women presumed to have been exposed to higher levels of testosterones *in utero* gave birth to significantly more male offspring [12].

Meanwhile, Helle & Lilley showed that maternal digit ratio was not a predictor of lifetime offspring sex ratio [19]. One possible answer is that Helle’s sample is of indirectly measured digit ratio and the repeatability of digit ratio measurement is very low (r = 0.79). Furthermore, Helle’s sample has also been used to refute a link between offspring sex ratio and age at menarche when it is likely that such a link exists. Fukuda et al. reported that women who had an early age at menarche tended to have an excess of daughters [60]. This suggests that early menarche should be linked to high digit ratio. This has indeed been reported, first by Matchock [61] and then in two large samples by Manning and Fink [62] and Kalichman et al. [63]. However, Helle [64] using the same sample of 241 postmenopausal Finnish women having a total of 494 singleton offspring as before reported no link between digit ratio and age at menarche. It has been suggested that Helle’s sample of Finnish women is small and atypical in at least some respects [65].

To date, the studies about the association between parental age and offspring sex ratio are controversial [66–68]. Our results showed that age was not related to digit ratio and offspring sex ratio (Table 2). However, in the present study, we did not investigate the patients’ age at the time of the birth. Actually, we investigated the patients’ age at the time of the investigation. Thus, we think that it is difficult to find the age effect on sex ratio in our data.

Birth data in Sweden for the period 1869–2004 showed that among live births the secondary sex ratio was on average 105.9 among singletons, 103.2 among twins and 99.1 among triplets [69]. This means that offspring sex ratio may be highest among singletons, medium among twins and lowest among triplets. However, in the present study, we investigated just the number of offspring. We did not distinguish multiples (twins and triplets) from singletons. In the present study, 77.6% (394/508) have two or more children (Table 6). As seen in the Table 6, offspring sex ratio decreases as the number of total offspring increases (Table 6). This result is similar to the study of Fellman and Eriksson [69]. Furthermore, when we re-analyzed on these 394 patients with two or more children, the results are similar to those of total 508 patients (Tables 7 and 8).

In the present study, we measured only the fingers of the right hand because there are numerous reports that differences between the sexes in digit ratio are greater on the right hand than on the left, and there are suggestions that the right hand may be more sensitive to the influence of testosterone [1,4,5,70–72]. In the present study, mean and median values of digit ratios of males, females, and all the patients are very close to 0.95. Therefore, we divided all the patients into two groups according to digit ratio cutoff of 0.95 (digit ratio < 0.95 versus digit ratio ≥ 0.95). Actually, when we divided the subjects according to median value of digit ratio in each sex, these results are similar to previous results with digit ratio cutoff of 0.95. Therefore, we think that digit ratio cutoff of 0.95 is appropriate. Additionally, in our previous studies, we also divided the subjects according to digit ratio cutoff of 0.95 [73–75].

Son preference and sex selective abortion have led to male-biased sex ratios in Asia, such as China, Korea, Vietnam, and India [76–87]. Even in this very low fertility society, such as Korea, son preference is a significant predictor of women's practice of induced abortion [83]. Thus, theses studies showed that induced abortion might be one of the evidences of artificial (man-made) sex selection. Therefore, it makes sense to exclude patients with a history of induced abortion that artificially affected the offspring sex ratio. However, spontaneous abortion, that is, intrauterine loss of fetuses might be one of the natural mechanisms that determine offspring sex ratio [88–92]. It has been suggested that stress exposure in early pregnancy can reduce male births via two mechanisms 1) reduced conception of males [93,94], and 2) increased intrauterine loss of male fetuses [89–92]. Therefore, we did not exclude patients with a history of spontaneous abortion.

### Limitations

The current study had some limitations. The participants were just recruited from among patients who were hospitalized for urological surgery at a single tertiary academic center and may therefore not accurately represent the general population. Nevertheless, we believe that our study produced sufficient evidence of a relationship between maternal digit ratio and offspring sex ratio since we excluded patients with a history of induced abortion that artificially affected the offspring sex ratio.

Another limitation of this study was that serum testosterone levels were not measured. We performed the present investigation based on the assumption that digit ratio is indicative of steroid hormone activity among individuals. If this assumption is correct, we think that our findings may reflect the relationship between steroid hormone activity among individuals and offspring sex ratio.

## Conclusions

In the present study, maternal (rather than paternal) digit ratio was negatively associated with offspring sex ratio. Females with a lower digit ratio were more likely to have more male offspring compared to those with a higher digit ratio. Overall, it could be suggested that the sex of offspring might be more influenced by maternal rather than paternal factors.

## Ethical Standards

Ethical approval (approval number: GBIRB2013-88) was obtained from the Institutional Review Board (IRB) of Gachon University Gil Hospital (Incheon, Republic of Korea). Our study was conducted according to the ethical principles laid down in the 1964 Declaration of Helsinki and its later amendments.

## Supporting Information

S1 Dataset Raw data of all 508 patients. Sex: 1 = male, 2 = female; Ht, Height; Wt, Weight; BMI, Body mass index; DR, Digit ratio; S, number of sons; D, number of daughters; T, number of total offsprings; SR, offspring sex ratio.
(XLS)
